# Supplementary material for: Comparison of electrophysiological left bundle branch pacing characteristics in different bilateral electrode pacing vector configurations
Source: Front Cardiovasc Med. 2025 Mar 12;12:1500196. doi: 10.3389/fcvm.2025.1500196 (PMC11936911; doi:10.3389/fcvm.2025.1500196)
Supplement: Supplementary Figure 1 — The changes in EGM and ECG during the threshold test of the three bilateral electrode pacing vector configurations. [file Image1.pdf]

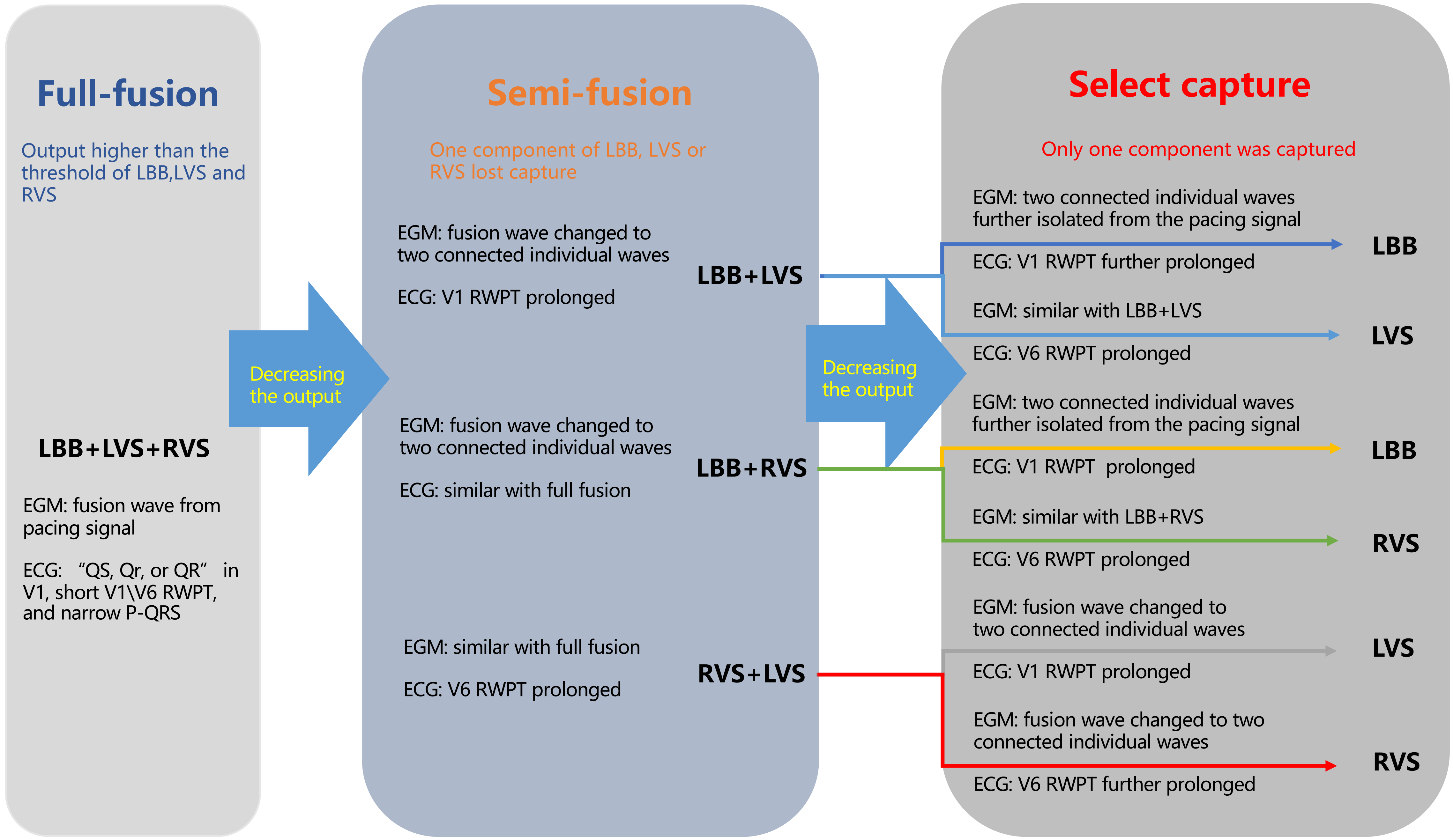
Full-fusion
Output higher than the threshold of LBB,LVS and RVS
LBB+LVS+RVS
EGM: fusion wave from pacing signal
ECG: "QS, Qr, or QR" in V1, short V1\V6 RWPT, and narrow P-QRS
Decreasing the output
Semi-fusion
One component of LBB, LVS or RVS lost capture
EGM: fusion wave changed to two connected individual waves
ECG: V1 RWPT prolonged
LBB+LVS
EGM: fusion wave changed to two connected individual waves
ECG: similar with full fusion
LBB+RVS
EGM: similar with full fusion
ECG: V6 RWPT prolonged
RVS+LVS
Decreasing the output
Select capture
Only one component was captured
EGM: two connected individual waves further isolated from the pacing signal
LBB
ECG: V1 RWPT further prolonged
EGM: similar with LBB+LVS
LVS
ECG: V6 RWPT prolonged
EGM: two connected individual waves further isolated from the pacing signal
LBB
ECG: V1 RWPT prolonged
EGM: similar with LBB+RVS
RVS
ECG: V6 RWPT prolonged
EGM: fusion wave changed to two connected individual waves
LVS
ECG: V1 RWPT prolonged
EGM: fusion wave changed to two connected individual waves
RVS
ECG: V6 RWPT further prolonged
